# MRI spectroscopic and tractography studies indicate consequences of long-term ketogenic diet

**DOI:** 10.1007/s00429-020-02111-9

**Published:** 2020-07-17

**Authors:** Kinga Gzieło, Krzysztof Janeczko, Władysław Węglarz, Krzysztof Jasiński, Krzysztof Kłodowski, Zuzanna Setkowicz

**Affiliations:** 1 Department of Neuroanatomy, Faculty of Biology, Gronostajowa 9, 30-387 Kraków, Poland; 2 Faculty of Physics and Applied Computer Science, AGH University of Science and Technology, 30-059 Kraków, Poland; 3 Department of Magnetic Resonance Imaging, Institute of Nuclear Physics Polish Academy of Sciences, Radzikowskiego 152 St, 31-342 Kraków, Poland

**Keywords:** Ketogenic diet, MRI spectroscopy, Normal rat, Tractography

## Abstract

To maintain its functional abilities, the mature brain obtains energy from glucose produced in carbohydrate metabolism. When carbohydrates are eliminated from the diet, the energy comes from the oxidation of fatty acids. In this metabolic state called ketosis, ketone bodies are formed: β-hydroxybutyric acid (bHb), acetone, and acetoacetate as alternative source of energy passing through the blood–brain barrier easily. The ketosis state can be achieved through various strategies like caloric restriction, supplementation with medium-chain triglycerides, intense physical training, or ketogenic diet (KD). Using KD, drug-resistant epilepsy has been successfully treated in children and adults. It can also exert neuroprotective influences in cases of brain damage, glioblastoma multiforme, and Alzheimer's or Parkinson's diseases. Although many possible mechanisms of KD activity have been proposed, newer hypotheses appear with the research progress, mostly characterizing the brain under pathological but not normal conditions. Since different pathological conditions may affect the mechanism of KD action differently, additional research on the normal brain appears reasonable. For this purpose, young adult rats were treated with 4-month-lasting KD. Then, MRI structural measurements, spectroscopy, and tractography were performed. The procedures revealed significant increases in the concentration of glutamine, glutamate, glutathione and NAA, accompanied by changes in the pattern of neuronal connections of the striatum and hippocampal formation. This implies a possible involvement of these structures in the functional changes occurring in the brain after KD application. Thus, the investigations on the normal brain add important details concerning mechanisms underlying KD effects without their possible modification by a pathological status.

## Introduction

Under normal circumstances, energy required for proper brain functioning in adults derives from glucose generated in carbohydrate metabolism (Vannucci and Vannucci 2000). When carbohydrates are eliminated from the diet, energy can be obtained from fatty acid oxidation. This process generates the amount of acetyl-co molecules sufficient for the synthesis of acetylacetic acid in the liver. It can be either spontaneously transformed into acetone or enzymatically transformed into beta-hydroxybutyric acid (ketone bodies, bHb). This reaction can run in both directions in the presence of beta-hydroxybutyric acid dehydrogenase (Bailey et al. 2005). Ketone bodies generated in this way are an alternative energy source for metabolism. The status in which the synthesis of ketone bodies is accelerated is called ketosis. Ketone bodies are transported to the circulation and then to extrahepatic tissues where they are used as ATP precursors. They can also reach the brain easily crossing the blood–brain barrier; acetone via simple diffusion, beta-hydroxybutyric acid and acetylacetic acid through transporters, the expression of which is connected with ketosis level (Bailey et al. 2005). Upon crossing the blood brain barrier, ketones are transported across cell plasma membranes via monocarboxylate transporters 1 and 2 in astrocytes and neurons, respectively (Vijay and Morris 2014).

Guzman and Blazquez (2001) presumed that ketone bodies might have a potential to arise also in astrocytes and then be transported into neurons. This astrocyte–neuron transportation hypothesis seems to be very interesting and worth studying in detail.

A diet with significantly reduced protein and carbohydrate is commonly called ketogenic (KD) and has been described by many authors as having protective effects. To date, several mechanisms of the ketogenic diet have been proposed, despite the fact that along with the increase of knowledge on this subject, the number of hypotheses has also been growing. It was also demonstrated that as short as a 3-week feeding of rats with KD gave rise to elevation of glutathione, a free radical scavenger. It was most probably associated with a simultaneous increase of glutamate cysteine ligase (GCL), an enzyme that limits the GSH synthesis rate (Jarrett et al. 2008).

Ketones may also affect the activity of sirtuin 3, which is the major mitochondrial deacetylase (Rardin et al. 2013). Due to its enhanced activity, the ROS production diminishes, thereby preventing from oxidative stress episodes. The ketogenic diet suppressed dissociation of a proapoptotic factor Bad from the chaperon protein 14-3-3 which was demonstrated to occur in the kainate model of seizures (Noh et al. 2005). Other studies showed that the ketogenic diet increased the level of the inhibitory neurotransmitter GABA and raised the expression of glutamic acid decarboxylase (GAD), an enzyme participating in GABA synthesis (Cheng et al. 2004; Dahlin et al. 2005; Yudkoff et al. 2005; Gama et al. 2015; Melo et al. 2006).

The neuroprotective potential of ketogenic diet related to epileptic seizures can be effective not only by changing levels of neurotransmitters, but also by independent modification of the neuronal membrane potential. After entering the TCA cycle, ketone bodies can increase ATP levels by affecting the ATP-dependent potassium channel and leading, therefore, to increased polarization of the neuronal membrane (Koppel and Swerdlow, 2017).

Recently, KD is frequently applied for the treatment of neurodegenerative diseases such as epilepsy, migraine, brain damage and Alzheimer’s or Parkinson's diseases (AD, PD), autism, sleep disorders, amyotrophic lateral sclerosis (ALS), multiple sclerosis (MS), pain, depression and even cancer (Stafstrom and Rho 2012) or to lose body weight. The reasons leading to the use of different dietary treatments as attempts to cure the diseases or alleviate their symptoms are both a lack of effective pharmacological treatment, and/or great needs for more natural methods without commonly occurring adverse side effects. Furthermore, it is believed that normally functioning neurometabolic paths can be modified by an appropriately selected diet (Stafstrom and Rho 2012).

However, the ameliorating KD effects were documented using mostly animal models or clinical trials on individuals whose metabolism was modified, or even disturbed by different damages or ongoing disease processes. Unlike those studies, our previous research on normal animals (Gzieło et al. 2019), which were not subjected to any previous experimental procedure, showed that KD led to morphological transformation of glial cells and quantitative increases of calretinin-containing interneurons. Using the same experimental design, our research is, therefore, aimed at effects of long-term, 4-month-lasting KD on brain anatomy (structural MRI scans), subtle changes in the brain metabolism (MRI spectroscopy), and intracerebral connections (MRI-based tractography). To our best knowledge, such studies have not been conducted so far. Only brain glucose and ketone uptake were tested in 4- or 21-month-old animals after 14-day-lasting KD (Roya et al. 2012). These studies showed only that the region-to-whole brain ratio of cerebral metabolic rate of glucose was 37–41% lower in the cortex and 40–45% lower in the cerebellum compared to cerebral metabolic rate of cerebral metabolic rate of acetoacetate in 4 month- and 21 month-old rats (Roya et al. 2012). In the study by Zhang et al. (2018a, b), NAA, Cho, CR contents were assessed with MRI spectroscopy in animals fed a ketogenic diet after TBI. In addition, studies by Huang et al. (2019) showed that KD applied for 3 months in mice did not cause changes in behavior when tested. But when the threshold for pentylenetetrazole-induced seizures was tested, it turned out to be higher in animals on KD.

## Materials and methods

### Animal treatment

In the experiment, we used twenty male Wistar rats from the Animal Clinic of the Pharmaceutical Department of the Jagiellonian University Medical College, Krakow. All procedures were carried out in accordance with the permission no. 122/2015 of the First Local Ethical Committee on Animal Testing at the Jagiellonian University in Krakow. At the age of 60 days, the rats were randomly divided into two equal groups and then subjected to MRI spectroscopy as described below. At the age of 65 days, in one of the animal groups, a ketogenic diet (ssniff^®^ EF R/M with 80% Fat, www.ssniff.com) was introduced, while the second group received a standard diet (Labofeed H Standard ND, Morawski). The animals on each type of diet were kept in individual cages for 4 months. The food consumed was weighed daily and the animals were weighed once a week. The composition of the feed in groups on each type is presented in Table 1.

**Table 1:** Percentage composition of diets used in the experiment

Nutrient	Ketogenic diet	Standard diet
Lipids	93	10
Carbohydrates	0	60
Proteins	6	30

Blood levels of glucose and ketone bodies were measured at the beginning of the diet administration and repeated every two weeks using a Optium Xido glucometer (Abbott Diabetes Care Ltd. Oxon, UK). After 4 months of treatments with these diets, MRI imaging, the same as that at the beginning of the experiment (spectroscopy), was repeated. Thereafter, the animals were anesthetized and venous blood was collected for peripheral blood count and biochemistry. Then perfusion-fixation was done with 3.7% formaldehyde in phosphate buffer, pH 7.4 (Setkowicz and Ciarach 2007; Gzielo et al. 2017). Brains and paratesticular fat were weighed. Finally, the brains were subjected to ex vivo MRI structural measurements and DTI (Diffusion Tensor Imaging).

### MRI spectroscopy before diet administration

**Fig. 1:**
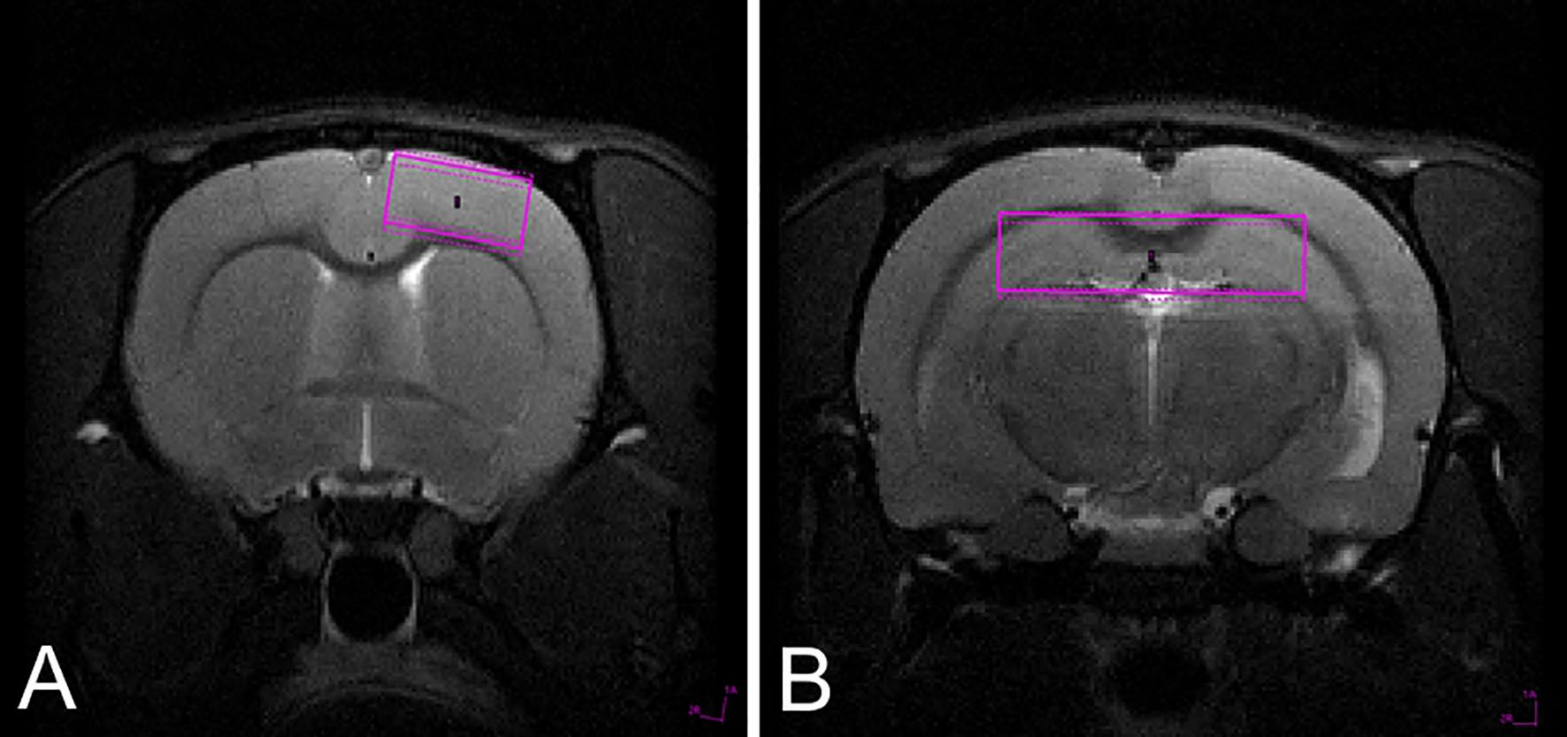
Position of spectroscopy voxel for signal acquisition from the cerebral cortex (**a**) and hippocampal formation (**b**)

The baseline concentrations of metabolites (Ala, Asp, Cr, PCr, GABA, Glc, Gln, Glu, GPC, PCh, GSH, Ins, Lac, NAA, NAA + NAAG, Cr + PCr, Glu + Gln, AcAc, Acn and bHb) scaled to the water signal were measured in single volume voxels in the cerebral cortex (1.8 × 4 × 4 mm size, Fig. 1a) and the hippocampal formation (2 × 8 × 2 mm size, Fig. 1b). Only metabolites with good LCmodel fit reliability (Cramer–Rao lower bound (CRLB) less than 20%) were included in further testing (Table 2).

**Fig. 2:**
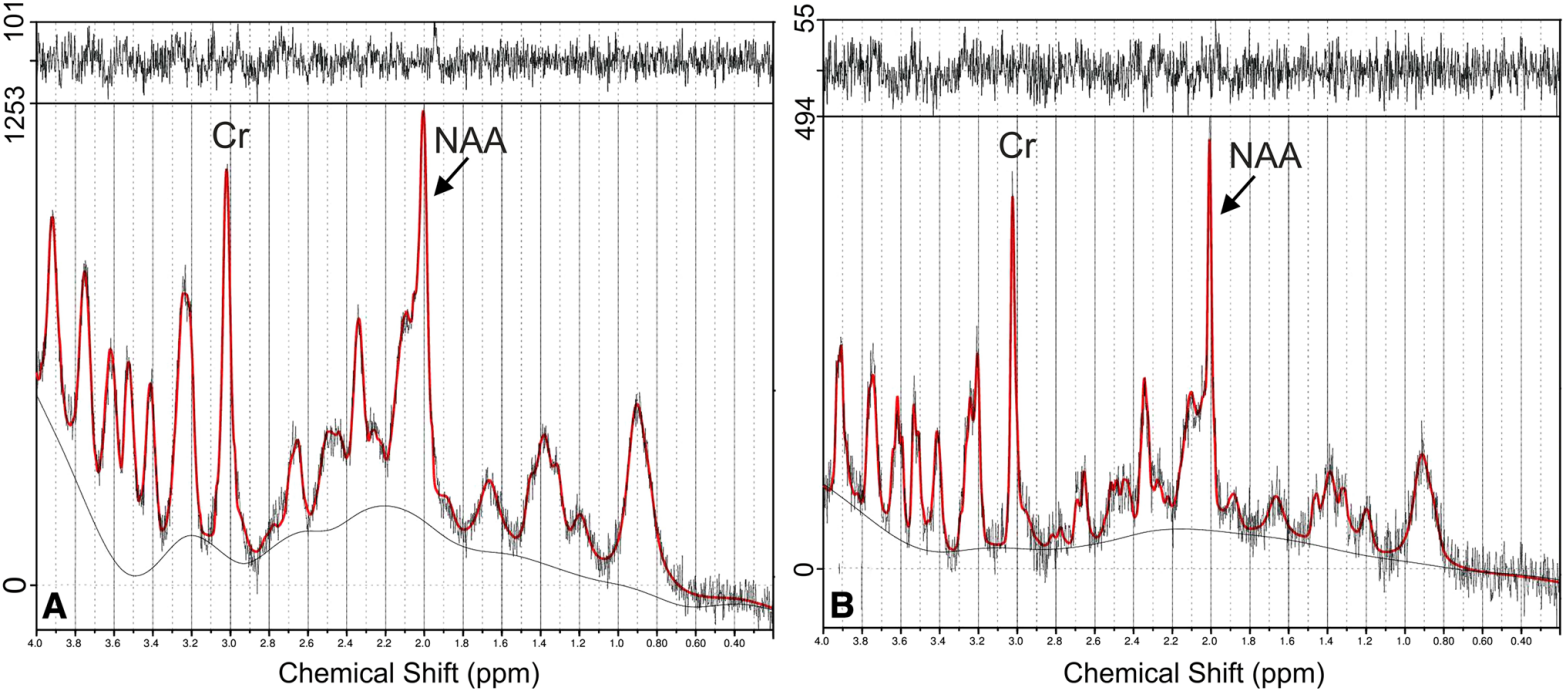
Spectra from the cerebral cortex in the same animal before (**a**) and after (**b**) ketogenic diet application with examples of relative concentrations shown for creatine (Cr) and *N*-acetylaspartate (NAA). The ordinate and abscissa show the relative concentration (signal intensity) and the frequency chemical shift (ppm), respectively. Note different scales of the relative concentration in Figs. A and B

**Table 2:** Metabolites analysed with MRI spectroscopy

Metabolite	Symbol
Acetone	Acn
Acetate	Act
l-Alanine	Ala
Aspartate	Asp
β-Hydroxybutyrate	bHb
Creatine	Cr
γ-Aminobutyric acid	GABA
Glucose	Glc
Glutamine	Gln
Glutamate	Glu
Glycerophosphocholine	GPC
Glutathione	GSH
myo-Inositol	Ins
l-Lactate	Lac
*N*-Acetylaspartate	NAA
*N*-Acetylaspartylglutamate	NAAG
Phosphocholine	PCh
Phosphocreatine	PCr
Taurine	Tau

### MRI spectroscopy after 4 months lasting diets

In animals that were on ND or KD, levels of Ala, Asp, Cr, Pcr, GABA, Glc, Gln, Glu, GPC, PCh, GSH, Ins, Lac, NAA, NAA + NAAG, Cr + PCr, Glu + Gln, AcAc, Acn and bHb were evaluated in the same brain areas in the cerebral cortex and hippocampal formation, similarly as at the initial stage of the experiment. The final levels of particular metabolites were compared with those recorded before the diet implementation, and between the ND and KD groups. Differences were tested for statistical significance by means* t* Student’s test for normal data distribution, *p* < 0.05 was considered statistically significant.

Magnetic resonance imaging and spectroscopy experiments were performed on a 9.4 T animal scanner (Biospec 94/20, Bruker, Ettlingen, Germany) running ParaVision 5.1. For in vivo measurements, BGA12S (Bruker) gradient system was used together with a quadrature transmit volume coil—T12054V3 (Bruker) and receive only rat brain surface coil—T11207V3 (Bruker).

Animals were placed in a water-heated rat bed with stereotactic fixation (Bruker) and nose cone to supply anesthesia. During the MRI, animal temperature, respiratory rate, and ECG were monitored with Small Animal Monitoring and Gating System, Model 1025 (SA Instruments, Inc. New York, USA). The animal temperature was kept between 37 and 38 °C and isoflurane (Aerrane, Baxter, Poland) concentration was 1.5% in a 1/2 oxygen/air mixture.

Based on reference multislice RARE scans (axial, sagittal and coronal), localized spectroscopy voxel was located in the hippocampal formation (Fig. 1b) and cerebral cortex (Fig. 1a). 1H MRS experiments were acquired using stimulated echo acquisition mode (STEAM). The acquisition parameters were as follows: echo time (TE) 3 ms, repetition time (TR) 2500, mixing time 10 ms, number of averages (NA) 700. The acquisition was triggered by animal respiration signal usually every second breath. VAPOR technique was used for water suppression and outer volume suppression (OVS) was applied as well. In case of hippocampal data acquisition, voxel size was 2 × 8 × 2 mm while for cerebral cortex it was 1.8 × 4 × 4 mm. Spectrum acquisition took usually 35 min. Reference scan without water suppression (RF power set to 0) was also acquired. Quantification of water-scaled metabolite concentration was performed using LCModel version 6.3-1M (Provencher 1999) program (Fig. 2).

### Ex-vivo MRI

This procedure was performed with a 35 mm inner diameter quadrature birdcage RF coil (Bruker T9913V3), BFG-113/60-S (Resonance Research, Inc. Billerica, MA, USA) gradient coil, and the ParaVision 6.1 software interface. For imaging, a fixed brain was enclosed in a syringe filled with formalin. DTI data were acquired with DtiStandard sequence [field of view (FOV) 18 × 18 mm, acquisition matrix (MTX) 128 × 128, slice thickness (SI) 0.5 mm, number of slices (NS) 46, repetition time (TR) 5000 ms, echo time (TE) 20 ms, number of averages (NEX) 4, 30 diffusion gradient directions, *b*-factor 1000 s/mm^2^, time between diffusion gradient pulses (Δ) 10 ms, diffusion gradient duration (*δ*) 2.5 ms]. Morphological T2 weighted images of the rat brain were acquired with RARE sequence [FOV 18 × 18 mm, MTX 256 × 256, SI 0.25 mm, NS 94, TR 3700 ms, TE 12.8 ms, turbo factor 4].

### Tractography

**Fig. 3:**
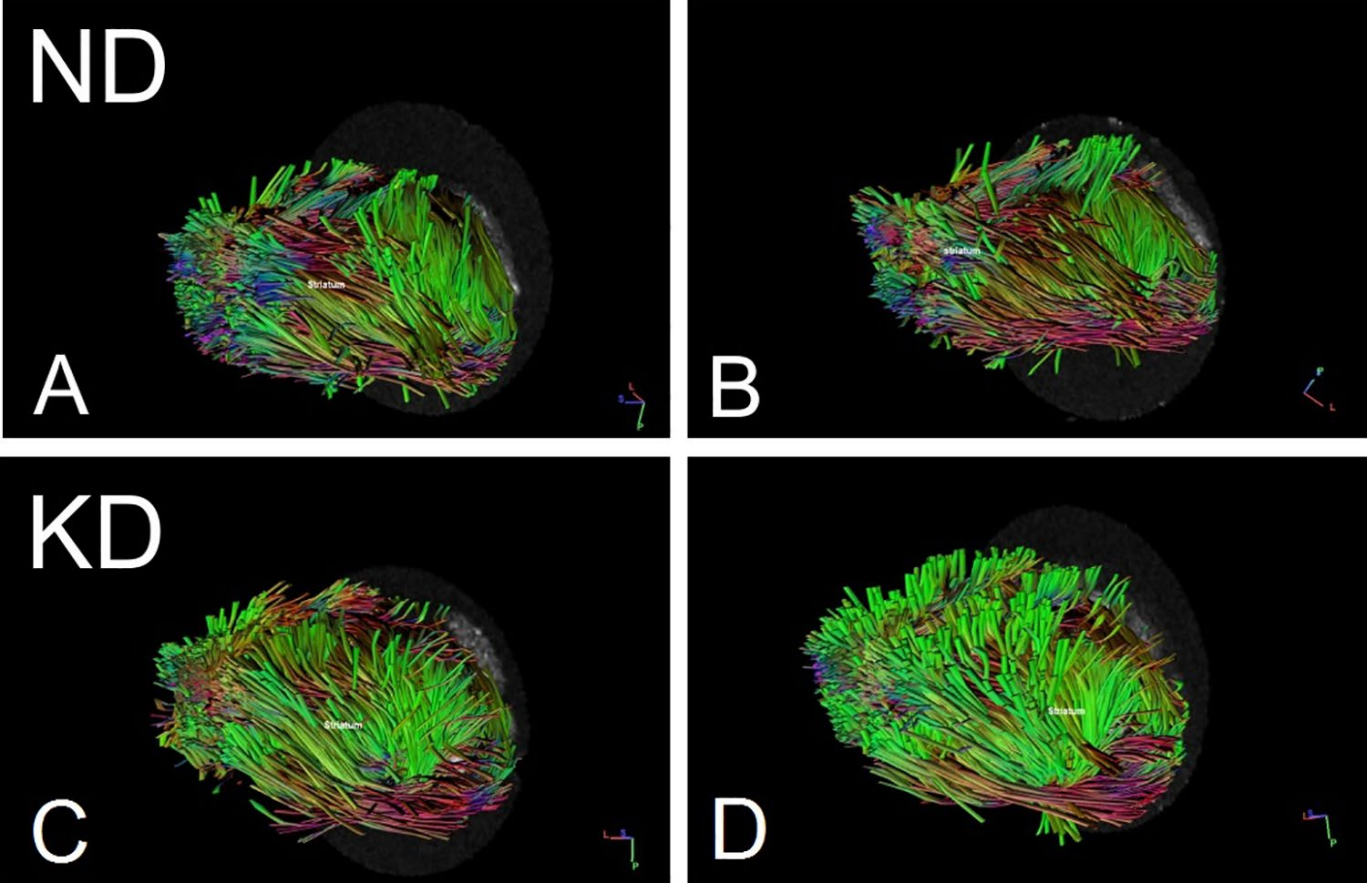
Examples of tractograms. Distribution of fibers belonging to the 3 + length class ending in the striatum in animals after 4-month-lasting normal (ND, **a** and **b**) or ketogenic (KD, **c** and **d**) diets. Fiber length classes are defined in “Results”

The tractography was obtained using DSI Studio (https://dsi-studio.labsolver.org). Diffusion MRI dataset was reconstructed with simple diffusion tensor model (DTI), giving high validation of connections (Maier-Hein et al. 2017). The deterministic streamline tracking algorithm was applied to the following regions selected manually in accordance to Waxholm Rat scalable brain atlas (Bakker et al. 2015; Papp et al. 2014; Kjonigsen et al. 2015; Sergejeva et al. 2015): anterior commissure, corpus callosum, fimbria-fornix, hippocampal formation, internal capsule, striatum and pons. The measured fibers were related to each of the specified brain regions and labeled here with respective symbols: (1) fibers go through the region of interest (ROI); (2) fibers do not go through that region (region of avoidance, ROA), (3) fibers enter that region and do not go further (END). Fractional anisotropy (FA) was used as a termination index with a threshold value equal to 0.15, angular threshold was set to 75 degrees, and the tracking algorithm was stopped after 20,000 seeds. Figure 3 shows examples of the obtained tractograms.

### Statistical analyses

Normality of data and homogeneity of variance were checked with the Shapiro–Wilk and Levene's tests, respectively. Because of normal distribution of the data, concentration levels of the examined metabolites were compared between groups using Student’s t tests for repeated measures or independent samples. Relationships between the concentration parameters were assessed by calculation of Pearson’s correlation coefficients. The level of statistical significance was set at 0.05. Statistical analyses were performed with Statistica 10 (Statsoft).

## Results

### Diet parameters

Measurements of food consumption per one animal showed that the average daily food intake was 20.0 g of normal (ND) and 10.5 g ketogenic diet (KD), providing 61.1 and 87.5 kcal/day, respectively.

It was observed that from the 11th week of experiment that the body weight of animals on KD was significantly higher than that of animals on ND (Gzielo et al. 2019). Despite that difference, very similar correlations were detected between the food intake and the weight of animals in each group (ND, *r* = 0.903, *p* < 0.000001; KD, *r* = 0.912, *p* < 0.00001).

### Blood parameters

**Fig. 4:**
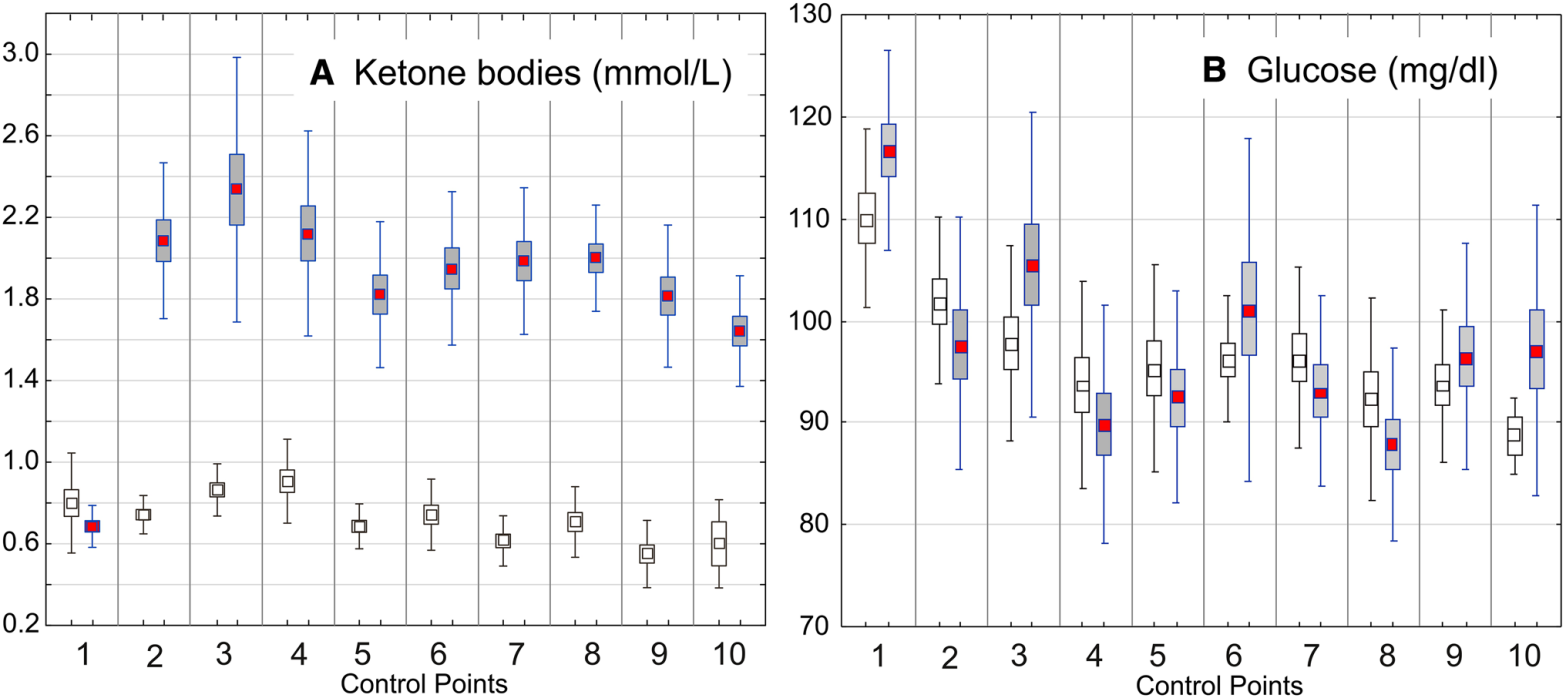
Blood levels of ketone bodies (**a**) and of glucose (**b**) measured at the beginning of the experiment (control point 1) then every 2 weeks (control points 2–10). In **a**, all the differences between KD-treated and control groups are statistically significant at least at *p* < 0.00001, with exception of that at the control point 1 representing the time when ketogenic diet was introduced (control point 1). The results are presented as means (± SEM and SD; Student’s *t *test) for animal groups on 4-month normal (white squares) or ketogenic diets (red squares), respectively

**Fig. 5:**
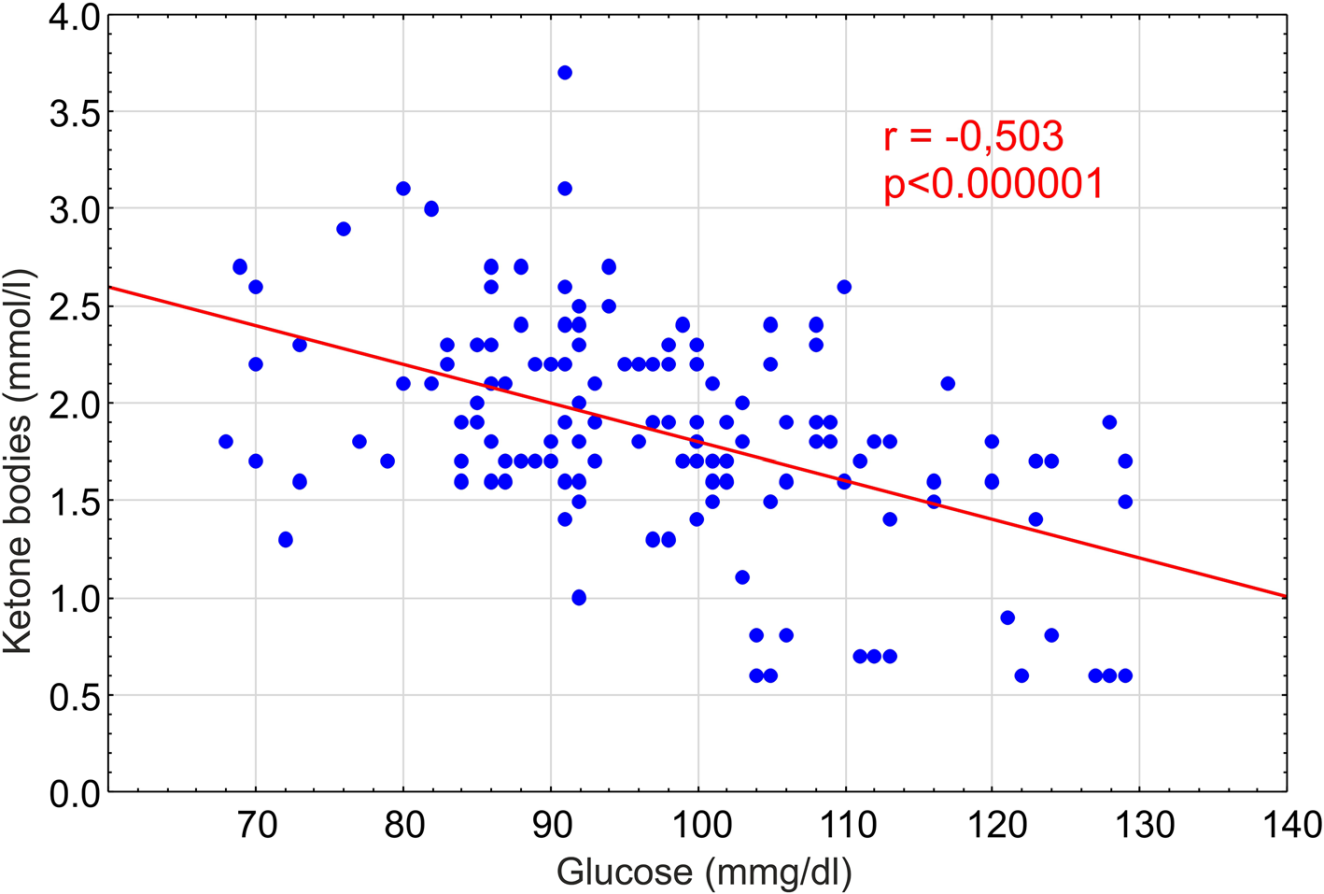
Correlation between blood levels of ketone bodies and glucose in rats on ketogenic diet. Pearson’s coefficient of correlation (*r*) is shown here with the index of statistical significance (*p*). A solid diagonal line shows a linear fit for the correlation

Ketone body blood concentration in animals at the second measurement (the first after the diet introduction) was significantly higher in animals on KD (Fig. 4). However, the average glucose level in all animal groups remained at 97 mM/L, but a significant correlation occurred between the ketone bodies and glucose levels in rats on KD (Fig. 5; *r* = − 0.503; *p* = 0.000001).

Before the end of the experiment (immediately before perfusion–fixation), the peripheral blood count was performed including HCT, HGB, MCH, MCHC, MCV, PLT, RBC, WBC without detection of a significant intergroup difference in the parameters tested. Biochemical blood tests of ALP, LDH, Kre, TG, CPK, UA detected statistically significant increases in ALP (alkaline phosphatase) (*p* < 0.0034) and TG (*p* < 0.0009) in the group on KD. In this group, the bHb level was also significantly increased (Gzielo et al. 2019).

### Metabolite concentration: MRI spectroscopy

**Fig. 6:**
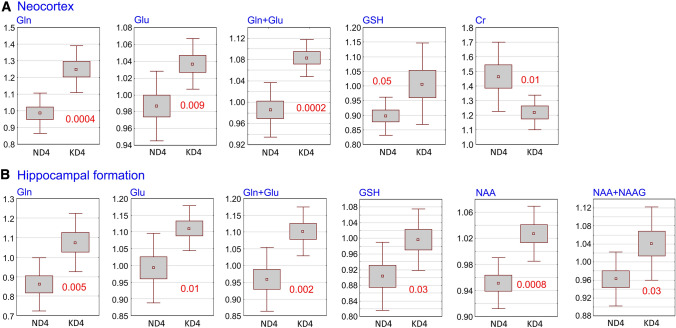
Changes in the concentration of selected metabolites in the cerebral cortex and hippocampal formation. The results are presented as means (± SEM and SD; Student’s *t *test) together with indexes of statistical significance (decimal fractions). ND4 and KD4—groups of animals on a 4-month normal or ketogenic diets, respectively. Abbreviations for metabolites are explained in “Materials and methods”

After 4 months of KD, significant increases in concentrations of Glu, Gln, Glu + Gln, and GSH were observed in the cerebral cortex with a decrease in Cr (Fig. 6a). In contrast, the concentrations of GABA, Glc, GPc, Ins, NAA, NAA + NAG, Pch, Pcr, Tau remained stable. The hippocampal formation of KD-treated animals, showed significantly increased concentrations of Gln, Glu, Glu + Gln, GSH, NAA and NAA + NAG. No changes in the concentration of GABA, Glc, Gpc, Ins, Pch, Tau, Ala, Lac were observed (Fig. 6b). Both the cerebral cortex and hippocampal formation showed no significant change of the ratio between Glu and GABA levels.

### Structural ex vivo MRI

On the MRI structural scans from brains of animals subjected to 4-month-lasting standard or ketogenic diets, volumes of the brain and of its regions related to the total brain volume were calculated. The measured regions were: the cerebral cortex, cerebral ventricles, hippocampal formation, striatum, midbrain and pons. The only statistically significant difference was that in the pons volume, which was significantly smaller in animals on KD (*p* < 0.04).

### Tractography

Based on ex vivo DTI fractional anisotropy measurements, myelinated fibers were divided into three classes, according to their minimum length, i.e. which were at least (i) 3 mm, (ii) 10 mm or (iii) 30 mm long. The classes are termed here as fibers 3+, 10+ or 30+, respectively. Myelinated fibers shorter than 3 mm were excluded from the analyses.

The DTI measurements revealed significant changes in the quantity, total length, total volume, and level of fractional anisotropy (FA) of fibers falling into each of these three classes and related to brain structures presented below (Table 3).

**Table 3:**
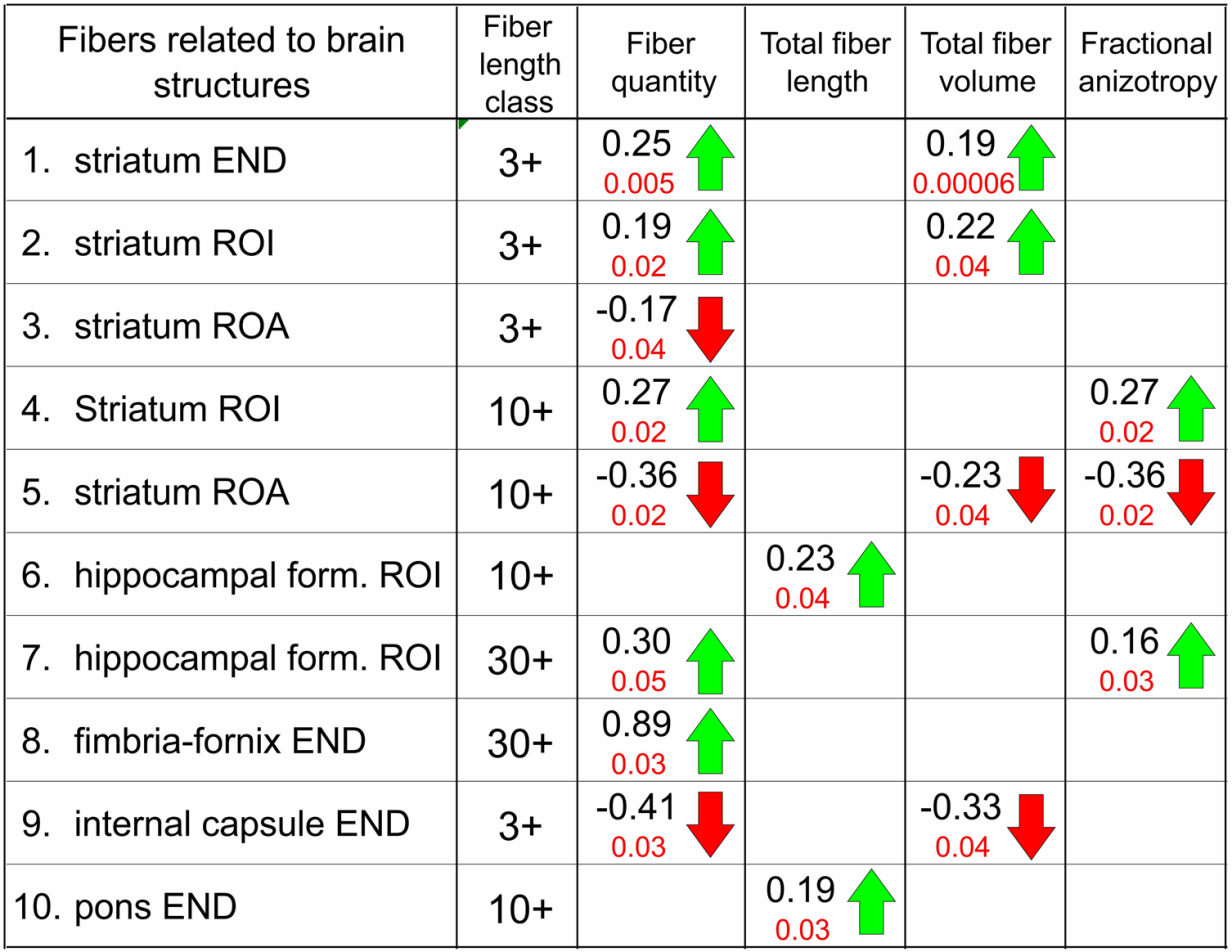
Distribution of myelinated fibers

Tractography analysis of myelinated fibers distribution was related to particular brain regions including the striatum, hippocampal formation, fimbria–fornix, internal capsule, and pons. Depending on their relations to brain regions, the fiber was defined as: ROI—fibers passing through a given region; ROA—fibers not passing through that region (region of avoidance); END—fibers entering but not leaving that region (end region). Fibers related to particular brain regions were characterized using four parameters: (1) total quantity, (2) total length, (3) total volume, and (4) fractional anisotropy. Symbols 3+, 10+and 30+ indicate length classes of fibers defined by their minimum length, i.e. which were at least (i) 3 mm, (ii) 10 mm or (iii) 30 mm long, respectively. Decimal coefficients (in black) show relative changes in values of particular parameters obtained by dividing differences between means from KD-treated (Mkd) and control groups (Mcon) by the mean from the control group (Mcon), i.e. using the following formula: (Mkd − MNcon)/Mcon. Increases and decreases are indicated by up- and down-oriented arrows (in green and red, respectively) with decimal indexes of statistical significance (Student’s *t* test for independent samples)

#### Striatum (Table 2/1–5)

The greatest changes in the distribution of myelinated nerve fibers occurred in relation to the striatum. Fibers 3+ mm ending in the striatum increased in their number (*p* < 0.005) and total volume (*p* < 0.00006). There were also increases in the number (*p* < 0.02) and volume (*p* < 0.04) of fibers 3 + mm passing through the striatum without ending in it. Respectively, the number of fibers 3+ mm that bypassed the striatum became significantly lower (*p* < 0.04). The number of fibers 10+ mm that passed through the striatum increased (*p* < 0.02), while the number (*p* < 0.02) and volume (*p* < 0.04) of those that bypassed the striatum (*p* < 0.02) decreased. This increase and decreases were correspondingly accompanied by an higher (*p* < 0.02) and a lower (*p* < 0.02) FA values.

#### Hippocampal formation of fimbria–fornix (Table 2/6–8)

The total length of fibers 3+ mm passing through this region increased (*p* < 0.04). Also numbers of fibers 30+ mm passing through the hippocampal formation increased (*p* < 0.05) with an elevation in their FA (*p* < 0.03). There was also an increase in the number of fibers 30+ mm ending in fimbria–fornix (*p* < 0.03).

#### Internal capsule (Table 2/9)

Significant decreases in the number (*p* < 0.03) and volume (*p* < 0.04) of fibers 3+ mm terminating in this region were detected.

#### Pons (Table 2/10)

Among all analyzed, only an increase in the total length of fibers 10+ was found as statistically significant (*p* < 0.03).

## Discussion

To our best knowledge, this is the first study on effects of long‐term feeding of KD in young adult rats, which were not subjected to any previous experimental procedure simulating a neurodegenerative disease. The study has shown that the 4-month-lasting ketogenic diet changed the metabolism and structure of the brain in normal, healthy animals. First, the changes were observed in the blood. As in other studies, after 1 week of KD consumption, the level of ketone bodies significantly increased in peripheral blood, which was the natural evidence of the treatment application. Also, in blood collected from the heart immediately before perfusion, the level of bHb was significantly higher (Gzieło et al. 2019). The glucose level remained normal, which was in agreement with Zhang et al. (2018a, b) who explained this fact as the result of adaptation to a long stay on a carbohydrate-free diet. Importantly, the morphotic blood elements were not affected, which suggests that these parameters were not modified when this diet was used in individuals suffering, for example, from epilepsy.

Since ketone bodies formed during the ketogenic diet can penetrate BBB, different parts of the central nervous system can also be affected but at different degrees. Our research was focused on changes in concentration of the most important brain metabolites occurring in the neocortex and hippocampal formation after 4 month-lasting KD. Level of the metabolites was measured with magnetic resonance spectroscopy frequently used not only for experimental approach but also in clinical diagnostic tests. Most of the metabolite levels did not change, which suggests that ketone bodies are rather not involved in all metabolic pathways within the nervous tissue.

The changes that we observed in both the cortex and hippocampal formation affected, among others, the levels of Glu and Gln which were significantly higher after 4-month-lasting KD than after normal diet. Glu is the most common excitatory neurotransmitter in the nervous system. Its occurrence and action are closely related to Gln via the Glu–Gln cycle. After being released from synapses, Glu is taken up by adjoining astrocytes, transformed into Gln, and then transported back to the neuron (Danbolt et al. 2016). That is why it was not surprising in our research that the increase in Glu after KD was accompanied by an increase in Gln level. The energetic cost of Glu uptake is very high—at 1.5 ATP molecules per one Glu molecule transported. Therefore, the large involvement of astrocytes in this process protects neurons from metabolic overload (Danbolt et al. 2016). For this uptake, KD provides more energy than a normal diet because metabolized fat delivers more energy than glucose (Włodarek 2019). Even though Glu is a precursor of GABA, in our study, the level of GABA did not undergo a corresponding KD-induced change. Also, no significant change was detected in the ratio of Glu to GABA concentration both in relation to the stage before KD introduction in the same animal group and to the control group fed with normal diet. Since GABA is the most important inhibitory neurotransmitter, increases in its level are postulated to determine epileptic seizure inhibition in response to KD (Yudkoff et al. 2008; Greene et al. 2003; Maalouf et al. 2009). In addition, Glu can act via ionotropic IGluRs receptors and enhance GABA secretion by inhibitory neurons (Mahmoud et al. 2019). However, KD does not always lead to increases in GABA content. As in our own study, KD-induced elevation of GABA did not occurr in the mouse forebrain and cerebellum (Yudkoff et al. 2001), mouse whole brain (Yudkoff et al. 2005), mouse neocortex (Melo et al. 2006) or in the whole rat brain (Hartman et al. 2007). Supposedly, the lack of changes in GABA concentration in our study resulted from the fact that we were focused on KD effects in normal animals but not in those under pathological conditions which can influence molecular interactions differently. This problem needs further investigations.

Another postulated mechanism of KD action is a decrease in oxygen free radical production. Such an antioxidant effect may be caused by increased levels of glutathione, which was also observed here. This is most likely associated with an increase in glutathione peroxidase activity. It is suggested that higher activity of this enzyme, induced by KD, may be protective against neurodegenerative changes (Ziegler et al. 2003). In addition, it is believed that most GSH-dependent detoxification processes occur in astrocytes (Fernandez-Fernandez et al. 2012), which confirms the involvement of these cells in the protective KD effects.

Our analyzes also showed a decrease in creatine concentration in the cerebral cortex. In our previous research using synchrotron radiation, an increase in the number of creatine deposits was detected in the hippocampal formation following 30-day-lasting KD (Skoczen et al. 2017). This discrepancy is probably due to different creatine content in particular brain structures. Creatine in the brain and its phosphorylated form acts as an energy reservoir necessary, among others, to maintain membrane potential (Kurosawa and Hamaoka 2017). Probably, during 4 months on KD, ketone bodies became the source of this energy, which is why in the cortex a decrease in the creatine content was observed while in the hippocampal formation it remained stable.

The above-mentioned changes in levels of metabolites associated with glial cells were accompanied by an increase in NAA found in the hippocampal formation. *N*-Acetylaspartate is one of the most common molecules in the brain. Its unique properties determine its extremely high concentration in the brain and its strong signal recorded with magnetic resonance spectroscopy (Setkowicz et al. 2015). Thus, it is represented by the largest peak in the spectrum of healthy brain tissue (Moffett et al. 2007) and its decrease is characteristic of many pathological conditions including, among others, different brain damages, ischemia, cancer, multiple sclerosis, or Alzheimer disease, (Danielsen and Ross 1999).

Despite the prevalence of NAA in the brain, its role remains not fully understood. It is thought to act as an osmolyte compensating anion deficiencies in neurons. It is considered as a precursor to NAAG dipeptide and the source of acetate necessary for the synthesis of myelin lipids. Supposedly, NAA supports the metabolism of neuronal mitochondria (Moffett et al. 2007). It is located in neurons. including their processes, at levels depending on specificity of a given neuronal population (Moffett et al. 2007). Besides that, strong interactions between glial cells and neurons mediated by NAA were detected. Baslow (2000) described this signaling pathway considering the fact that both NAA and NAAG are synthesized in neurons while the related catabolic enzymes are found in oligodendrocytes and astrocytes.

Therefore, NAA released from neurons may be a signal for oligodendrocytes and NAAG for astrocytes (Moffett et al. 2007). Then, after completing the appropriate catabolic reactions in target cells, the substrates can be reutilized by neurons for NAA and NAAG resynthesis. It can be concluded that the elevation of NAA and NAAG contents following KD is a natural functional consequence of presence of NAA as the source of acetoacetate for the production of myelin. The number of mitochondria and levels of their metabolism increase (Hartman et al. 2007), so it seems to lead to higher concentration of NAA, which is involved in energy processes in mitochondria. Thus, the supply of substrates necessary for the myelination process increases, affecting particular brain regions differently.

Following the high fat diet conditions in our investigation, the striatum, as the biggest brain region of interest, showed the most spectacular signs of increases in myelinated fibers. These increases were accompanied by declines in parameters for fibers bypassing the striatum and other ending in the internal capsule. It can be assumed that the two fiber components might, at least partially, overlap each other and form a part of connections with the neocortex. The striatum controls motor functions through brainstem structures and pathways passing through the pons. Thus, the relatively small increases in the pons volume and in the length of 10+ fibers terminating in it could have been associated with the increases found in the striatum. The hippocampal formation and fimbria–fornix, belonging to the same functional subsystem, show consistent increases in 3+ and 30+ fibers which may have functional effects.

All these results indicate that KD could evoke significant changes in intracerebral connections but, because of their preliminary and general character, particular fiber components cannot be recognized.

The effect of ketogenic diet on the intracerebral connectivity has not yet been studied. Our tractography measurements revealed the strongest significant increases related to the striatum, the area responsible for animal movement. Available data on the effect of ketogenic diet on locomotor activity is ambiguous (Murphy et al. 2005; Murphy and Burnham 2006; Thio et al. 2010), and those on the motor system in the normal brain are very rare. So far, KD effects have been reported in diseases with substantial motor dysfunctions and in corresponding rodent models of Alzheimer's and Parkinson’s diseases, amyotrophic lateral sclerosis, or spinal cord injury (Veyrat-Durebex et al. 2018). Little is known about the molecular mechanisms underlying observed improvements, especially when only a selective local impact on the striatum would be considered, including associated increases in the fiber quantity. The subject of our research was the impact of KD on the normal brain, and this means the absence of a pathological condition that can significantly modify the consequences of KD.

In KD-treated rats, an elevated expression of glutamate decarboxylase was detected in the striatum (Cheng et al. (2004). This could be indirect evidence of functional changes in this brain region since this enzyme is involved in GABA synthesis. In the present study, we found quantitative increases in fibers related not only with the striatum but also with the hippocampal formation and fornix. Selective KD effects on these regions, but not on the cerebral cortex, were reported by Zarnowski et al. (2012) but concerning increased levels of kynurenic acid (KA). Similarly to the present study, this regional effect was detected in normal animals without any extra pathological influences. However, Dabrowski et al. (2015) reported that, in the spinal cord, myelin was negatively affected by elevated KA concentration, so it could rather not promote increases in fiber quantities like those in our study. The very diversified results obtained for different regions of the central nervous system might also indicate possible differences between local responses to KD action (Cheng et al., 2004) or even between responses within the same region (Balietti et al. 2008). This might also depend on changes in a local functional status or the type of neuronal subsystems within a given structure. Therefore, these results need more detailed examinations.

## Conclusions

The present study showed that KD applied to normal animals had a little effect on general brain anatomy, detected only as a volumetric increase of the pons. However, significant changes occurred in the pattern of intracerebral connections. They were most strongly related to the striatum and appeared to point to changes in the role of this region in the entire neural network of the brain. At the same time, as the result or cause of these changes, there were increases in the concentrations of metabolites being of critical functional importance for the nervous system such as NAA, glutamate or glutathione. Since the most significant changes were related to metabolites associated with glial cells, especially astrocytes, it can be assumed that these cells are the main target of KD and can also underlie its neuroprotective effects. Confirmation requires further research.

## Abbreviations

Acn: Acetone
Act: Acetate
Ala: l-Alanine
ALP: Alkaline phosphatase
Asp: Aspartate
ATP: Adenosine triphosphate
bHb: β-Hydroxybutyric acid
CPK: Creatine phosphokinase
Cr: Creatine
FA: Fractional anisotropy
GABA: γ-Aminobutyric acid
GAD: Glutamic acid decarboxylase
GCL: Glutamate cysteine ligase
Glc: Glucose
Gln: Glutamine
Glu: Glutamate
GPC: Glycerophosphocholine
GSH: Glutathione
HCT: Hematocrit
HGB: Hemoglobin
Ins: Myo-Inositol
Lac: l-Lactate
LDH: Lactate dehydrogenase
MCH: Mean corpuscular hemoglobin
MCHC: Mean corpuscular hemoglobin concentration
MCV: Mean corpuscular volume
NAA: *N*-Acetylaspartate
NAAG: *N*-Acetylaspartylglutamate
PCh: Phosphocholine
Pcr: Phosphocreatine
PLT: Blood platelets
RBC: Red blood cells
ROS: Reactive oxygen species
Tau: Taurine
TCA: Citric acid cycle
TG: Trigliceryde
UA: Uric acid
WBC: White blood cells
